# Genetic analysis of hantaviruses carried by *Myodes *and *Microtus *rodents in Buryatia

**DOI:** 10.1186/1743-422X-5-4

**Published:** 2008-01-11

**Authors:** Angelina Plyusnina, Juha Laakkonen, Jukka Niemimaa, Kirill Nemirov, Galina Muruyeva, Boshikto Pohodiev, Åke Lundkvist, Antti Vaheri, Heikki Henttonen, Olli Vapalahti, Alexander Plyusnin

**Affiliations:** 1 Department of Virology, Haartman Institute, University of Helsinki, Finland; 2 Finnish Forest Research Institute, Vantaa, Finland; 3 Swedish Institute for Infectious Disease Control, Stockholm, Sweden; 4 Buryat State Academy of Agriculture, Ulan-Ude, Buryatia, Russian Federation; 5 Department of Basic Veterinary Medicine, University of Helsinki, Finland

## Abstract

Hantavirus genome sequences were recovered from tissue samples of *Myodes rufocanus, Microtus fortis *and *Microtus oeconomus *captured in the Baikal area of Buryatia, Russian Federation. Genetic analysis of S- and M-segment sequences of Buryatian hantavirus strains showed that *Myodes-*associated strains belong to Hokkaido virus (HOKV) type while *Microtus-*associated strains belong to Vladivostok virus (VLAV) type. On phylogenetic trees Buryatian HOKV strains were clustered together with *M. rufocanus- *originated strains from Japan, China and Far-East Russia (Primorsky region). Buryatian *Microtus- *originated strains shared a common recent ancestor with *M. fortis- *originated VLAV strain from Far-East Russia (Vladivostok area). Our data (i) confirm that *M. rufocanus *carries a hantavirus which is similar to but distinct from both Puumala virus carried by *M. glareolus *and Muju virus associated with *M. regulus*, (ii) confirm that *M. fortis *is the natural host for VLAV, and (iii) suggest *M. oeconomus *as an alternative host for VLAV.

## Background

Hantaviruses (genus *Hantavirus*, family *Bunyaviridae*) are negative-strand RNA viruses with a tripartite genome, each carried by a specific rodent or insectivore host [1]. Some hantaviruses, e. g. Hantaan and Seoul viruses in Asia, Puumala (PUUV), Dobrava and Saaremaa viruses in Europe, Sin Nombre and Andes viruses in the Americas, are human pathogens while others, e.g. *Microtus-*associated hantaviruses of both hemispheres are considered apathogenic [2,3]. For some hantaviruses, e.g. PUUV-like Hokkaido virus (HOKV) associated with *Myodes rufocanus *or Topografov virus (TOPV) carried by *Lemmus sibiricus*, pathogenicity was neither convincingly demonstrated nor completely ruled out [4,5].

In addition to the abovementioned HOKV, TOPV, Hantaan, and Seoul viruses, several more hantaviruses have been found in Asia. These include three well-established species: Thottapalayam virus in *Suncus murinus *[6], Thailand virus in *Bandicuta indica *[7], and Khabarovsk virus (KHAV) in *M. fortis *[8]. Also several provisional species have been described: Da Bie Shan virus in *Nivivevnter confucianus *[9], Gou virus in *Rattus rattus *[9], Vladivostok virus (VLAV) in *M. fortis *[10], Amur/Soochong virus in *Apodemus peninsulae *[11,12], and Muju virus (MUJV) in *Myodes regulus *[13]. Of all Asian hantaviruses, so far only TOPV was found in Siberia. In this project we attempted to analyze hantaviruses circulating in Buryatia, the autonomy in Russian Federation located between the Lake Baikal and Mongolia. The biogeographic position of Buryatia is interesting because the taiga corridor zone south of the Lake Baikal has been important for the exchange of eastern and western elements of the palearctic fauna.

## Methods

### Screening of rodent samples

Rodents were trapped in August 2005 in five localities in Buryatia, Russian Federation. Samples of 504 small mammals were collected, including samples from lung, kidney and spleen (of most animals) in RNA *Later *[Ambion] and in Laemmli sample buffer, and in addition a blood sample dried on filter paper. The blood samples were extracted from the filter paper to PBS and were screened by immunofluorescence assay (IFA) for the presence of antibodies to hantaviruses (Puumala and Dobrava virus antigens) (the details of trapping and IFA-screening will be published elsewhere). Ab-positive rodents were checked for the presence of hantaviral N-antigen (N-Ag) using immunoblotting of the lung tissue samples as described earlier [14,15].

### RNA isolation, reverse transcription (RT)-polymerase chain reaction (PCR) and sequencing

RNA was purified from N-Ag-positive lung tissue samples with the TriPure reagent (Boehringer Mannheim), according to the manufacturer's instructions. RNA was then subjected to the RT-PCR to recover: (i) complete or partial (coding region) S segment sequences, and (ii) partial (nt 2766-3007) M segment sequences (sequences of primers and other experimental details are available upon request). PCR amplicons have been gel-purified with QIAquick Gel Extraction-kit (QIAGEN) and sequenced either directly or after cloning into pGEM-T vector (Promega) using ABI PRISM™ Dye Terminator or ABI PRISM™ M13F and M13R Dye Primer sequencing kits (PerkinElmer/ABI, NJ), respectively. HOKV genome sequences described in this paper have been deposited to the GenBank sequences database under accession numbers AM930972, AM930975, and AM930976. VLAV genome sequences described in this paper have been deposited to the GenBank sequences database under accession numbers AM930973, AM930974, AM930977, AM930978, and AM930979.

### Phylogenetic analysis

To infer phylogenies, the PHYLIP program package [16] was used. Hantavirus sequences used for comparison were recovered from the GenBank. 500 bootstrap replicates generated for complete coding sequences of the S segment, as well as partial sequences of the M segment (Seqboot program) were submitted to the distance matrice algorithm (Dnadist). Distance matrices were analyzed with the Fitch-Margoliash tree-fitting algorithm (Fitch) or with Neighbor-joining algorithm (Neighbor) using the ML model for nucleotide substitutions; the bootstrap support values were calculated with the Consense program. The nucleotide sequence data were also analyzed using the Tree-Puzzle program (maximum likelihood) [17] with the HKY model for nucleotide substitutions and 10 000 puzzling steps.

## Results

### Screening of rodent samples for the presence of hantaviral markers

Rodents were first screened by IFA for the presence of anti-hantaviral antibodies. Altogether eight Ab-positive rodents were selected; these were further checked for the presence of hantaviral N-Ag. Five animals were found positive, namely two *Myodes rufocanus*, #767 and #791, captured near Muhorshibir town, one *Microtus oeconomus*, #483 captured near Barguzin river and two *Microtus fortis*, #500 and #503, trapped in the vicinity of Nesterikha village. All five N-Ag-positive rodents were analyzed by RT-PCR with hantavirus-specific primers and all five were found positive for hantaviral RNA. Hantaviral S and M segment sequences were recovered from these five rodents. Complete S segment sequences were recovered from *M. rufocanus *#767, *M. oeconomus *#483, and *M. fortis *#503. Partial S segment sequences were recovered from *M. rufocanus *#791 (almost complete coding region) and *M. fortis *#500 (complete coding region). These partial sequences appeared identical to the corresponding parts of the S-sequences recovered from *M. rufocanus *#767 and *M. fortis *#503, respectively. Partial M segment sequences were recovered for all five animals: nt 2766-3007 for *Microtus*, and nt 2702-3007 for *Myodes *(the numeration is given for PUUV sequence).

As expected, S- and/or M-sequences from *M. rufocanus *showed the closest similarity to the previously described *M. rufocanus- *originated sequences from Japan (Hokkaido), China (Fusong) and Far-East Russia (Primorsky region). The name "Hokkaido virus (HOKV)" has been suggested for the *Myodes *(erlier called, *Clethrionomys*) *rufocanus- *associated hantavirus and, following this line, we designated Buryatian wild-type (wt) strains as HOK/Muhorshibir/Mr767/2005 and HOK/Muhorshibir/Mr791/2005, or Muhorshibir767 and Muhorshibir791, for short.

The S-sequences from Buryatian *M. fortis *showed the closest similarity to *M. fortis-*originated sequence recovered from Vladivostok area, Far-East Russia [10] thus providing additional evidence that this rodent species can harbor VLAV. We designated these hantavirus strains VLA/Nesterikha/Mf503/2005, and VLA/Nesterikha/Mf500/2005 or Nesterikha503 and Nesterikha500, for short. To our surprise, the S-sequence recovered from *M. oeconomus*, captured near river Barguzin, was very close to the S-sequence of Nesterikha503 strain. To rule out possible mistakes in species identification, mtDNA from rodent #483 was analyzed and its initial identification as *M. oeconomus *confirmed. Since hantavirus genome sequence recovered from *M. oeconomus *belonged to VLAV genotype the corresponding wt hantavirus strain was designated as VLA/Barguzin/Mo483/2005, or Barguzin483, for short.

### Genetic analysis of Buryatian HOKV strains

**Figure 1:**
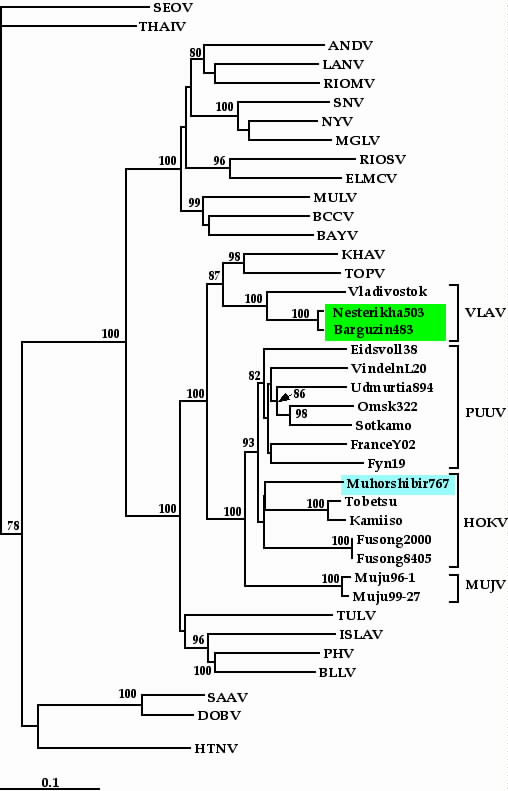
Phylogenetic tree (Fitch-Margoliash) of hantaviruses based on the complete coding region of the S segment. Only bootstrap support values greater than 70% are shown. HTNV, Hantaan virus, strain 76–118; DOBV, Dobrava virus (strain Slovenia); SAAV, Saaremaa virus, strain Saaremaa; SEOV, Seoul virus strain (R22); THAI, Thailand virus strain Thailand/Bi50); ANDV, Andes virus (strain Chile9717869); LANV, Laguna Negra virus (strain 510B); RIOMV, Rio Mamore virus (strain OM556); SNV, Sin Nombre virus (strain NMH10); NYV, New York virus (strain New York-1); MGLV, Monongahela virus (strain Monongahela1); RIOSV, Rio Segundo virus (strain RMx-Costa-1); ELMCV, El Moro Canyon virus (strain RM97); MULV, Muleshoe virus (strain SH-Tx-339); BCCV, Black Creek Canal virus; KHAV, Khabarovsk virus (strain MF43); TOPV, Topografov virus (strain TopografovLs136); VLAV, Vladivostok virus; PUUV, Puumala virus; HOKV, Hokkaido virus; MUJV, Muju virus; TULV, Tula virus (strain Moravia02); ISLAV, Isla Vista virus (strain MC-SB-47); PHV, Prospect Hill virus (strain PH1); BLLV, Blood Land Lake virus (strain Mo46).

As mentioned above, the S segment sequence of Muhorshibir767 strain appeared most closely related to the corresponding sequences of other HOKV strains. For coding regions the sequence identity was 84–86% (3'-noncoding regions could not be aligned unequivocally). On the phylogenetic tree calculated for the S segment coding region (Fig. 1), strain Muhorshibir767 was placed within the well supported clade formed by three hantavirus species associated with *Myodes *voles: PUUV, HOKV, and MUJV. Within this clade, PUUV and HOKV shared a more ancient common ancestor and the pair appeared as sister taxa to MUJV. Notably, the deduced amino acid (aa) sequences of the N protein of PUUV, HOKV, and MUJV were of the same length (433 aa residues), a clear sign of their close genetic ties and shared evolution. Furthermore, the N protein sequence of Muhorshibir767 strain not only showed the highest identity to the corresponding sequences of HOKV strains from Hokkaido and Fusong (97.5% and 96.5%, respectively) but also carried a specific aa signature of HOKV: Val/Ile68, Ile262. In addition, all HOKV strains shared seven signature aa residues with PUUV: Asp6, Ile134, Val251, Ser305, Ala313, Ile324, and Gly/Ser412, but only four with MUJV: Lys5, Arg26, Lys258, and Pro283. On the S-tree (Fig. 1), HOKV strains formed three genetic lineages represented, respectively, by strains from Japan (strains Kamiiso and Tobetsu), China (Fusong strains), and Buryatia (strain Muhorshibir767). It should be noted that, while PUUV strains (the ones shown on the Fig. 1 belonged to six genetic lineages) formed a distinct, well-supported group, the monophyly of HOKV strains did not receive a substantial bootstrap support. Hopefully, when more HOKV sequences become available the phylogenetic resolution would improve.

**Figure 2:**
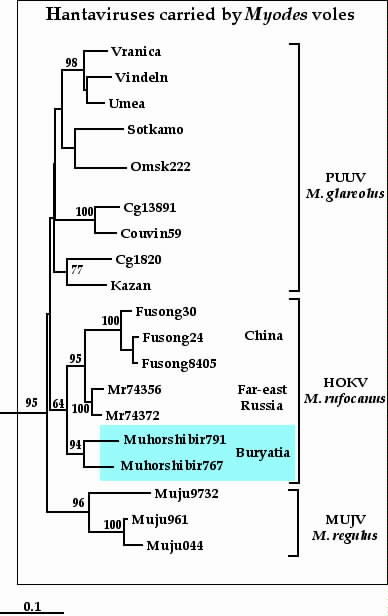
Phylogenetic tree (Fitch-Margoliash) of *Myodes*-associated hantaviruses based on partial M segment sequence (nt 2702-3007). For abbreviations, see Fig. 1.

Similarly to the S segment sequences, the M-sequences recovered from Buryatian *M. rufocanus *were most closely related to the corresponding sequences of other HOKV strains: the ones from China (Fusong) [sequences available from GenBank] and also Far-East Russia (Primorsky region) [18]. The M segment sequences of Japanese strains were not determined yet and, consequently, the Japanese genetic lineage was not presented on the phylogenetic tree (Fig. 2). Within HOKV group, Chinese and Russian strains showed geographical clustering and formed a distinct genetic lineage. Another lineage was formed by two Buryatian strains. The monophyly of all HOKV strains was supported reasonably well (64%). Again, all *Myodes- *originated sequences showed the host-dependent clustering of PUUV, HOKV and MUJV genetic variants, in good agreement with the idea of co-evolution of hantaviruses with their natural carriers [19-21].

### Genetic analysis of Buryatian VLAV strains

The S segment sequences of two Nesterikha strains were identical; their partial M-sequences differed by one silent substitution only (sequence identity 99.6%). Complete S-sequences of strains Nesterikha503 and Barguzin483 were very close to each other: only nine nucleotide substitutions were found in the coding region (sequence identity 99.3%) and only seven mutations, six substitutions and one deletion, in the 3'-noncoding region that, in this case, could be easily aligned. Seven of nine nucleotide substitutions found in the coding region were silent. Two mutations caused homologous aa substitutions in the deduced sequence of the N protein: Lys96->Arg and Ile326->Val. Partial M segment sequences of these two strains differed by three silent substitutions (sequence identity 99.2%).

**Figure 3:**
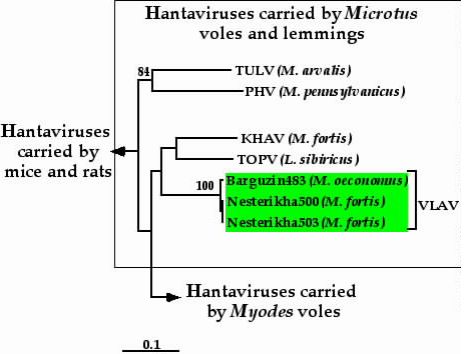
Phylogenetic tree (Fitch-Margoliash) of *Microtus*-associated hantaviruses based on partial M segment sequence (nt 2766-3007). For abbreviations, see Fig. 1.

Two closely related S segment sequences of Buryatian VLAV strains showed the highest level of sequence identity to the prototype wt strain from Vladivostok (Far-East Russia): 87.3–87.4%. The deduced aa sequences of the N protein of two Buryatian VLAV strain were 95.5% identical to the corresponding sequence of Vladivostok strain. The N-sequence identities with closely related KHAV and TOPV were substantially lower: 91.0% and 90.3%, respectively. On the phylogenetic tree constructed for the S segment coding region (Fig. 1), two Buryatian VLAV strains clustered in the closest proximity to each other and shared the most recent common ancestor with the Vladivostok strain. This trio, in turn, shared a more ancient common ancestor with KHAV and TOPV. All the branches within this group were well supported (87% to 100%). Phylogenetic trees calculated for partial M segment sequences showed a similar branching pattern (Fig. 3). All three VLAV strains from Buryatia clustered together and formed a monophyletic group with KHAV and TOPV. However, the bootstrap support values were, again, rather low, most probably due to the very small number (and, perhaps, also the low genetic diversity) of the sequences available for this analysis.

## Discussion

There are no earlier data on hantaviruses in Buryatia. The taiga region south of Lake Baikal has acted as a corridor for the palearctic fauna and Buryatian rodents were therefore of special interest in the study of hantavirus evolution in Eurasia. *Myodes rufocanus *is found throughout the palearctic taiga, and *Microtus fortis *is at the northwest corner of its distribution range in Buryatia. Samples of both species were found positive in our immunoblotting assay; this suggested a possible involvement of HOKV and KHAV or VLAV. Earlier, HOKV genome sequences were recovered from *M. rufocanus *captured in Japan, Far-East Russia and China [10,18]. *M. fortis *was known as the natural host for KHAV [8]. Our earlier analysis of KHAV and TOPV suggested that a host-switch had occurred in the evolution of these hantaviruses [5].

There was also a report describing the recovery of partial hantaviral S segment sequence of from *M. fortis *captured near Vladivostok [10]. In our experiments, RT-PCR followed by sequencing confirmed the presence of HOKV and VLAV in *M. rufocanus *and *M. fortis*, respectively. These data support the notion on *M. fortis *as a natural host for VLAV. Furthermore, our findings of VLAV sequences in *M. oeconomus *suggest that this rodent species could carry VLAV as well. Notably, both vole species belong to the same subgenus *Alexandromys *in the genus *Microtus*, i.e. genetically they are closely related to each other [22]. Of course, spillover of VLAV from its real host (whatever it is) to other sympatric rodent species, cannot be excluded and therefore further investigation of this issue is needed.

Phylogenetic analysis of newly recovered Buryatian hantavirus sequences was complicated by limited datasets available for HOKV and especially VLAV genetic variants. This, in our opinion, was the very reason for the lower than desired resolution (seen as <70% bootstrap support values for a number of branching points). Our previous experience tells that, at least in some cases, an addition of one-two "critical" sequences to the dataset could remarkably improve the phylogenetic resolution [23,24]. Some improvement could also be achieved by the recovery of longer M segment sequences directly from rodent tissue samples. So far, this presented a real problem for our Buryatian collection. Isolation of HOKV and VLAV in cell culture would, undoubtedly, speed the progress in this direction. Despite these drawbacks, the general phylogeny of HOKV genetic variants looked logical and supportive to the hypothesis of hantavirus-host co-evolution (Fig. 1 and Fig. 2).

Our finding of VLAV sequences in *M. oeconomus *was a bit surprising and thus added a new twist to the already quite intriguing relationships between TOPV, KHAV, and VLAV. KHAV and VLAV, both carried by *Microtus *voles, do not cluster on the phylogenetic trees with other hantaviruses carried by *Microtus *(TULV, PHV, ISLAV, and BLLV) but instead appeared monophyletic with TOPV and the KHAV-VLAV-TOPV trio forms a sister taxon to the *Myodes*-carried hantaviruses: PUUV, HOKV, and MUJV (Fig. 1). This clustering is in apparent disagreement with strict hantavirus-host co-evolution and would suggest host-switching event(s). One could imagine three possible scenarios. The first scenario includes a host-switch of an ancient hantavirus from *Myodes *to *Lemmus *yielding an ancestor for TOPV, KHAV, and VLAV, followed by two independent, and separated in time, host-switching events of a *Lemmus-*associated virus to *Microtus *yielding KHAV and VLAV. The second scenario involves a "jump" of an ancient hantavirus from *Myodes *to *Microtus *followed by diversification of hosts for KHAV and VLAV and another "jump" of pre-KHAV from *Microtus *to *Lemmus *yielding TOPV. The third scenario implies that *Microtus-*associated hantaviruses are the most ancestral ones among the group carried by *Arvicolinae *rodents. According to this scenario, in a single host-switching event an ancient *Microtus-*carried virus gave origin to the ancestor of all *Myodes-*carried viruses and later pre-KHAV virus "jumped" from *Microtus *to *Lemmus*, producing TOPV.

Future studies, incuding analysis of larger sets of TOPV, KHAV and VLAV variants, preferably originated from a wider geographical area and showing substantial genetic diversity, would be needed to evaluate these hypotheses.

## Conclusion

In this paper, for the first time, we describe HOKV and VLAV strains found in Buryatia, Russian Federation. Although no human cases have been so far attributed to either of these two hantaviruses, further epidemiological studies are needed to estimate a seroprevalence to HOKV and VLAV (as well as other hantaviruses, such as Amur/Soochong virus carried by *Apodemus peninsulae*) in Buryatian population and to evaluate potential threats to human health which might be imposed by these hantaviruses.

## Competing interests

The author(s) declare that they have no competing interests.

## Authors' contributions

AngP participated in the screening of rodent samples, performed RT-PCR and sequencing, participated in the genetic analysis and contributed to writing of the manuscript. JL, JN and BP participated in fieldwork and in screening of rodent samples. KN performed genetic analysis of rodents. GM and ÅL participated in the study coordination. AV, HH and OV participated in the study design and coordination, and in drafting the manuscript, the latter two also in fieldwork. AP participated in the study design and coordination, performed (phylo)genetic analyses and contributed to writing of the manuscript. All authors read and approved the final manuscript.
